# Extreme Delta Brush in Anti-NMDAR Encephalitis Correlates With Poor Functional Outcome and Death

**DOI:** 10.3389/fneur.2021.686521

**Published:** 2021-07-09

**Authors:** Nabeela Nathoo, Dustin Anderson, Jeffrey Jirsch

**Affiliations:** ^1^Division of Neurology, Department of Medicine, University of Alberta, Edmonton, AB, Canada; ^2^Department of Critical Care, University of Alberta, Edmonton, AB, Canada

**Keywords:** anti-NMDA receptor encephalitis, EEG, extreme delta brush, ICU, neurocritical care

## Abstract

**Objective:** To characterize EEG findings in anti-NMDAR encephalitis patients looking for the proportion of EEGs that were abnormal, presence of extreme delta brush (EDB), and to relate EEG findings to clinical outcomes (Glasgow Outcome Scale (GOS) at 6 months, need for ICU admission, and death).

**Methods:** This retrospective cohort single center study included patients with anti-NMDAR encephalitis who had ≥1 EEGs obtained from 2014 to 2021. EEGs were retrospectively analyzed by 2 reviewers. Clinical outcomes of interest were extracted through hospital and clinic chart review.

**Results:** Twenty-one patients with anti-NMDAR encephalitis were included. Sixty-four EEGs were analyzed. Four EEGs (6.3%) were within normal limits. Focal or generalized slowing (without EDB) was seen on 44 EEGs (68.8%). EDB was seen on 16 EEGs (25.0%) in 9 of 21 patients (42.9%). The presence of EDB was significantly associated with need for ICU admission (*p* = 0.02), poorer outcome at 6 months as per the GOS (*p* = 0.002), and with death (*p*=0.02). EDB was present on ≥1 EEG of every patient who died.

**Conclusions:** The presence of EDB on EEG in anti-NMDAR encephalitis patients is associated with increased need for ICU admission, worse functional outcomes at 6 months, and risk of death.

## Introduction

Anti-N-methyl-D-aspartate receptor (NMDAR) encephalitis is an immune-mediated encephalitis that can be paraneoplastic to an ovarian teratoma. Clinical presentation involves some combination of subacute behavioral change including, but not limited to hallucinations and delusions; seizure; profound dysautonomia; and hyperkinetic movement disorders including oromotor dyskinesias, chorea, and dystonia, but can be challenging to diagnose early (1, 2). Early identification is critical as it can lead to prompt treatment resulting in better outcomes (2, 3). Among the para-clinical tests used to aid in the diagnosis of anti-NMDAR encephalitis, electroencephalography (EEG) is included in the diagnostic criteria and is often helpful as it is rarely normal (4). Furthermore, the EEG pattern extreme delta brush (EDB) is thought to be highly specific to anti-NMDAR encephalitis (5). Resembling waveforms seen in premature infants, EDB is a distinctive pattern of synchronous and symmetric 1–3 Hz transients with superimposed bursts of rhythmic 12–30-Hz activity present almost continuously in an EEG recording (5).

Although EEG is helpful in diagnosing anti-NMDAR encephalitis, the specific EEG patterns seen, evolution of EEG findings during the disease course, and the prognostic value of certain EEG findings like EDB remain incompletely understood. In this study, we characterized EEG findings in a cohort of anti-NMDAR encephalitis patients from our local center.

## Methods

### Subjects

This was a single center retrospective cohort study taking place at the University of Alberta between January 2014 and February 2021. Pediatric and adult patients with definite anti-NMDAR encephalitis (confirmed by the presence of anti-NMDAR antibodies in CSF) who had undergone at least one EEG within 1 month of diagnosis were included. Testing sites for anti-NMDAR antibodies in CSF were the Mayo Clinic (Rochester, Minnesota, USA) and Mitogen Lab (Calgary, Alberta, Canada). Information on clinical outcomes for patients was obtained retrospectively through hospital and clinic chart review. Glasgow Outcome Scale (GOS) scores (6) were estimated based on clinical documentation from follow-up 6 months after CSF anti-NMDAR antibody testing was positive. This study was approved by the University of Alberta Research Ethics Office.

### EEG Analysis

Two reviewers (DA, JJ) retrospectively analyzed all EEGs of anti-NMDAR patients independently that were undertaken at the University of Alberta Hospital. In patients whom more than one EEG was obtained, all EEGs obtained within 6 months of CSF anti-NMDAR antibodies being positive were evaluated. EEGs were analyzed with attention to the following: presence or absence of a posterior dominant rhythm (PDR), presence of pathological slowing (diffuse vs. focal, theta vs. delta range), electrographic seizures, interictal epileptiform discharges, and presence of EDB. The method of Schmitt et al. (5) was used to identify EDB: for a day's EEG recording a nearly continuous combination of delta activity with superimposed fast activity, usually in the beta range, most often symmetric and synchronous, typically seen broadly across all head regions, not varying with sleep-wake cycles and not varying significantly with stimulation or level of arousal.

### Statistical Analyses

GraphPad Prism 9.0.2 was used for statistical analyses. Contingency analysis looking at whether the presence of EDB was significantly associated with status (alive vs. deceased), need for ICU admission, and GOS score (good outcome being a score of 4 or 5, poor outcome being a score of 1–3), being female, and presence of a teratoma was conducted using Fisher's exact test (two-sided). The same analyses for status, need for ICU admission, and GOS score were carried out separately using only the first EEG. Comparisons for the number of EEGs obtained and age of patients in those with EDB vs. without EDB were carried out using unpaired *t*-tests. A *p*-value of <0.05 was deemed to be statistically significant.

## Results

A total of 21 patients with definite anti-NMDAR encephalitis were included. Demographics of the patient population are shown (Table 1). Patients were aged 11–60 (mean age 26.6 ± 12.2 years) with 5 of 21 (23.8%) under age 18. Four of 21 patients (19.0%) died, all of whom were over age 18. A range of 1–7 EEGs were obtained for each patient (mean 3.0 ± 1.7). Two pediatric patients had a period of continuous EEG monitoring.

**Table 1:** Patient demographics and EEG findings for 21 subjects with anti-NMDAR encephalitis.

	**Pediatric (*n* = 5)**	**Adult (*n* = 16)**	**Total (*n* = 21)**
Age, median (range)	13 (11–17)	28 (18–60)	26 (11–60)
Female, n (%)	3 (60)	14 (87.5)	17 (81)
Teratoma present, n (%)	0 (0)	10 (62.5)	10 (47.6)
ICU admission, n (%)	1 (20)	11 (68.8)	12 (57.1)
Death, n (%)	0 (0)	4 (25)	4 (19)
Glasgow Outcome Scale score of 1–3 at 6 months, n (%)^*^	1 (25)	6 (43.9)	7 (38.9)
Glasgow Outcome Scale score of 4–5 at 6 months, n (%)^*^	3 (75)	8 (57.1)	11 (61.1)
All EEGs normal, n (%)	2 (40)	0 (0)	2 (9.5)
At least 1 EEG with slowing, n (%)	3 (60)	16 (100)	19 (90.5)
At least 1 EEG with extreme delta brush, n (%)	2 (40)	7 (43.8)	9 (42.9)

* *GOS is not reported for 3 patients as they are fewer than 6 months out from anti-NMDAR encephalitis diagnosis*.

Sixty-four EEGs were analyzed, of which 4 (6.3%) were within normal limits. In 3 of 21 patients (14.3%), the first EEG was within normal limits; in 2 of those 3 patients, the second EEGs were abnormal. Thus, only 1 patient in our cohort had only normal EEGs. The most common abnormal EEG finding was that of focal or generalized slowing (without EDB), seen in 44 EEGs (68.8%). Slowing was both asymmetric and symmetric, often in the delta range.

**Figure 1:**
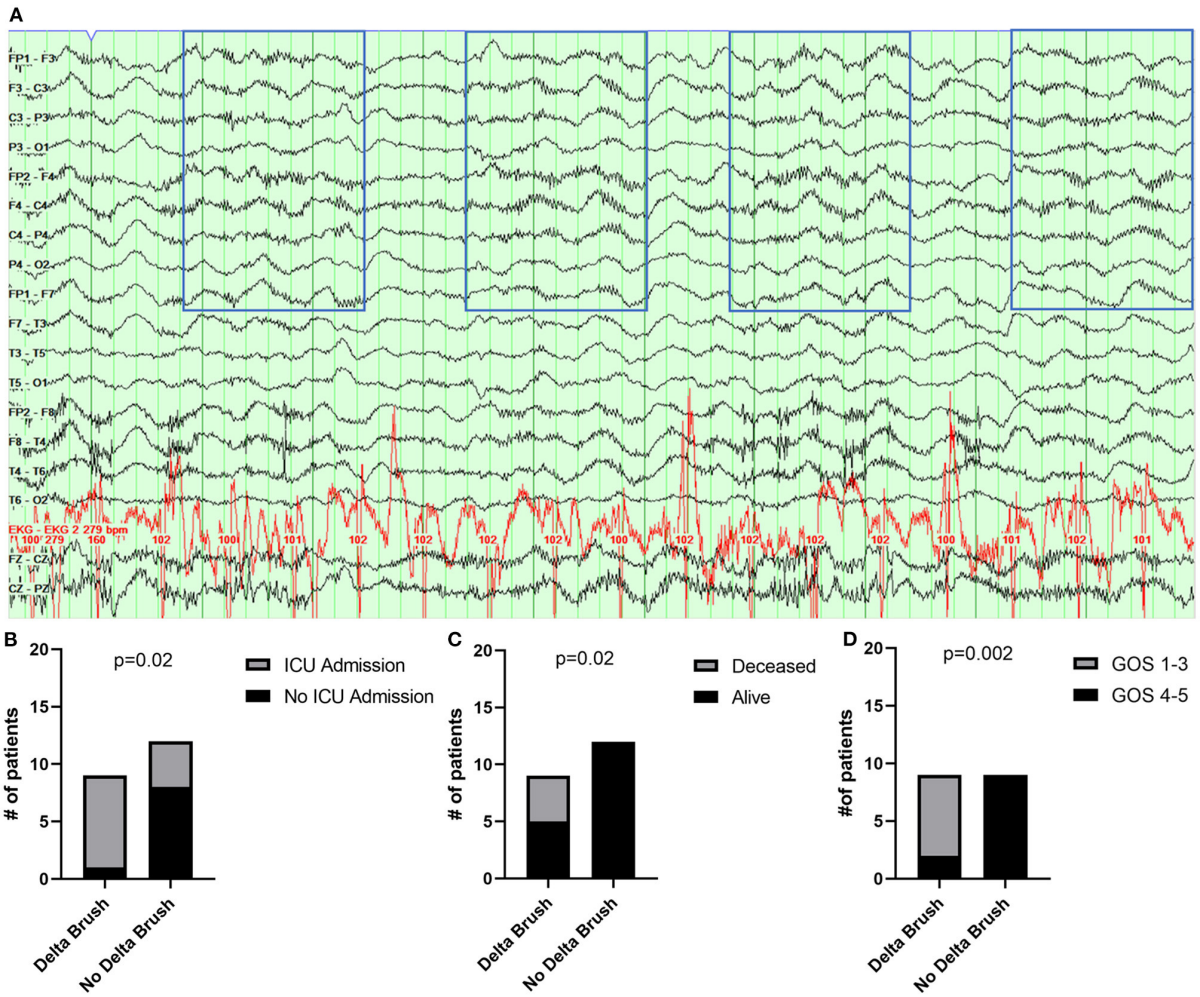
Association between extreme delta brush and need for ICU admission, death, and functional outcome. **(A)** Extreme delta brush (EDB) is seen (outlined by blue boxes) on this bipolar montage EEG in a patient with anti-NMDAR encephalitis. **(B)** Association between EDB and need for ICU admission. **(C)** Association between EDB and status (alive vs. deceased). **(D)** Association between EDB and GOS score at 6 months (score of 1–3 indicative of poor outcome, score of 4–5 indicative of good outcome).

Characteristics of patients with EDB compared to those without EDB on EEG are shown (Table 2). EDB (Figure 1A) was present on 16 EEGs (25.0%) in 9 of 21 patients (42.9%); it was present in 2 of 5 (40%) of the pediatric patients. In 5 of 9 patients, EDB was not seen on the first EEG but was seen on a subsequent EEG (2 on the second EEG, 2 on the third EEG, 1 on the fourth EEG). In 8 of 9 patients, EDB resolved on subsequent EEGs. Once EDB resolved, it did not recur on a future EEG. Eight of 9 patients (88.9%) with EDB required ICU admission, compared to 4/12 (33.3%) without EDB (*p* = 0.02) (Figure 1B, Table 2). Notably, EDB was present in at least one EEG of every patient in our cohort who died, which was 4 of the 9 patients total with EDB (44.4%), compared to 0/12 without EDB (*p* = 0.02) (Figure 1C, Table 2). Of the 5 patients who had EDB that remained alive, 4 were under the age of 20, and the fifth patient had a protracted course requiring first, second, and third-line immunosuppression, and underwent empiric oophorectomy with an occult teratoma (7). EDB on EEG was associated with poorer functional outcome as measured by the GOS with 7/9 (77.8%) with EDB having a GOS of 1-3, compared to 0/12 without EDB having a GOS of 1–3 (*p* = 0.002) (Figure 1D, Table 2). Three patients (1 pediatric, 2 adult) were excluded from GOS analysis as they were diagnosed fewer than 6 months ago. The aforementioned relationships between EDB and ICU admission, death, and functional outcome with GOS were not statistically significant when only the first EEG obtained was analyzed (*p* > 0.05 for all).

**Table 2:** Patient demographics in those with anti-NMDAR encephalitis with and without extreme delta brush on EEG.

	**EDB (*n* = 9)**	**No EDB (*n* = 12)**	***p*-value**
Age, mean ± standard deviation	22.9 ± 7.7	29.4 ± 14.5	0.24
Female, n (%)	8 (88.9)	9 (75)	0.60
Teratoma present, n (%)	4 (44.4)	6 (50)	>0.99
ICU admission, n (%)	8 (88.9)	4 (33.3)	**0.02**
Death, n (%)	4 (44.4)	0 (0)	**0.02**
Glasgow Outcome Scale score of 1–3 at 6 months, n (%)^*^	7 (77.8)	0 (0)	**0.002**
Glasgow Outcome Scale score of 4–5 at 6 months, n (%)^*^	2 (22.2)	9 (100)	
Number of EEGs obtained, mean ± standard deviation	3.8 ± 1.5	2.5 ± 1.7	0.09

* *GOS is not reported for 3 patients as they are fewer than 6 months out from anti-NMDAR encephalitis diagnosis*.

*The bold values indicate statistically significant relationships as the p-values are less than 0.05*.

## Discussion

In our cohort study, the great majority of EEGs recorded at the time of the patients' anti-NMDAR encephalitis were abnormal. Most patients had non-specific focal or generalized slowing on their EEGs, with EDB present in less than half of cases. This study's finding that EDB, when present, was associated with poorer functional outcomes of patients at 6 months and with death has not previously been reported to our knowledge.

In our study, over 90% of patients' EEGs were abnormal around the time of their acute illness. Our study aligns with others' that found 96% of EEGs in their cohort were abnormal (8) and a systematic review looking at EEG findings in anti-NMDAR encephalitis where over 80% of EEGs were abnormal (4). A number of abnormalities have been described on EEGs in this patient population, EDB being only one example (9).

We saw EDB on EEG in 42.9% of patients analyzed in our study. This aligns closely with the first descriptions of EDB in anti-NMDAR encephalitis where authors noted that 30% of patients had this “unique” finding (5). Nearly half of the pediatric patients in our cohort had EDB, comparable to 53% that has been reported by others (10). Although less common than focal or generalized slowing, identifying EDB is important as it is associated with more prolonged hospitalization, (5) being refractory to first-line immunotherapy, (11) and the need for ICU admission (5) which our study aligns with. Every patient in our cohort who died had EDB on either their first EEG or a later EEG, and the presence of EDB was significantly associated with risk of death. Most who did survive with EDB on EEG were under the age of 20 and therefore the significance of the EEG finding in pediatric cases is less clear.

The underlying pathophysiology of EDB is incompletely understood. Some insight has been gleaned with dynamic causal modeling showing that in cases of anti-NMDAR encephalitis that there are persistent baseline changes that make cortical microcircuits sensitive to fluctuations in synaptic coupling which may lead to paroxysmal abnormalities, like EDB (12). Another study using dynamic causal modeling with a microcircuit model showed alteration of the network involving fronto-parietal regions in anti-NMDAR encephalitis (13). Altogether, studies conducted so far suggest that changes occurring at the synaptic level can lead to network level changes with altered synchronization, and thus, paroxysmal changes on EEG in anti-NMDAR encephalitis.

In our study, only 10% of patients underwent continuous EEG monitoring and EEG findings (including the presence of EDB) were detected mostly using repeated inpatient 45–60 min recordings as the disease evolved. However, some patients had only a single EEG obtained and so findings of significance (i.e., EDB) may have been missed in some of our cases. The exact sensitivity of detecting EDB on first EEG is unknown. In one cohort study, EDB was seen in 6% of patients in the first EEG but in subsequent EEG in 20% of patients (8). However, serial EEGs are more likely to be obtained in more unwell patients in the critical care setting, which increases the likelihood of detecting EDB. This is a potential source of bias in detection of EDB which should be kept in mind. Lastly, EEGs were not obtained consistently after immunotherapy, precluding us from commenting on how EEG findings changed after treatment.

This study highlights the importance of obtaining serial EEGs (or carrying out continuous EEG) in anti-NMDAR encephalitis patients with special attention looking for EDB as it can be associated with an increased risk of death in adults. Outcomes specifically looking at death should be included in future analyses of EEG findings in anti-NMDAR encephalitis.

## Data Availability Statement

The original contributions presented in the study are included in the article/supplementary material, further inquiries can be directed to the corresponding author.

## Ethics Statement

The studies involving human participants were reviewed and approved by University of Alberta Research Ethics Office. Written informed consent to participate in this study was provided by the participants' legal guardian/next of kin.

## Author Contributions

NN designed and conceptualized the study, analyzed and interpreted the data, drafted, and revised the manuscript for intellectual content. DA designed and conceptualized the study, played a major role in acquisition of the data, analyzed the data, and revised the manuscript for intellectual content. JJ designed and conceptualized the study, analyzed the data, and revised the manuscript for intellectual content. All authors contributed to the article and approved the submitted version.

## Conflict of Interest

The authors declare that the research was conducted in the absence of any commercial or financial relationships that could be construed as a potential conflict of interest.
